# Effect of Maternal Administration of Edible Bird's Nest on the Learning and Memory Abilities of Suckling Offspring in Mice

**DOI:** 10.1155/2018/7697261

**Published:** 2018-03-22

**Authors:** Yong Xie, Hongliang Zeng, Zhiji Huang, Hui Xu, Qunyan Fan, Yi Zhang, Baodong Zheng

**Affiliations:** ^1^College of Pharmacy, Fujian University of Traditional Chinese Medicine, Fuzhou 350122, China; ^2^College of Food Science and Technology, Fujian Agriculture and Forestry University, Fuzhou 350002, China; ^3^Xiamen Silk Food Co. Ltd, Xiamen 361100, China

## Abstract

Although human brains continue developing throughout the underage developmental stages, the infancy period is considered the most important one for the whole life. It has been reported that sialic acid from edible bird's nest (EBN) can facilitate the development of brain and intelligence. In this study, by oral administration of EBN to female mice during the pregnancy or lactation period, the effects of EBN on the levels of sialic acid in mouse milk were determined using high-performance liquid chromatography (HPLC). Furthermore, the spatial learning performances of their offspring were assessed using the Morris water maze test. Additionally, cerebral malondialdehyde (MDA), superoxide dismutase (SOD), choline acetyltransferase (ChAT), and acetylcholinesterase (AChE) in cubs nursed by the female mice given the EBN homogenate were examined, while BDNF immunohistochemical staining and neuron count in hippocampi were investigated as well. These results showed that administration with EBN in maternal mice during pregnancy or lactation period can improve the learning and memory functions in their offspring, possibly by increasing the activities of SOD and ChAT and, at the meantime, decreasing the levels of MDA and activities of AChE. Moreover, BDNF levels for CA1, CA2, and CA3 regions in hippocampi and the numbers of dyed neurons in CA1, CA2, CA3, and DG regions among the offspring were significantly enhanced due to the intake of EBN by the maternal mice. We concluded that maternal administration of EBN during the pregnancy and lactation periods can improve the spatial learning performances in the offspring.

## 1. Introduction

Human intelligence is a complex genetic trait that rests on the interactions between a multitude of genes and certain environmental factors (e.g., diet, drugs, and stress) experienced by parents. The brain development in human is a very complicated process, during which the infancy period is recognized as the most important one. In this period, breast milk is the primary nutrition source and the ideal food that infants can obtain [1]. The studies on the rodents have proved that, along with the brain development, the learning and memory functions are continuously being developed. Meanwhile, the hippocampus plays an important role in memory functions, emotional behavior, processing of novel information, or integrating social information [2].

EBN is a kind of nest which is made by male swiftlet mainly through its saliva during breeding stage. It has been collected and used as both cuisine and pharmacy for thousands of years [3, 4]. According to the ancient records [5], consumption of EBN can be traced back to Tang dynasty (618–907 AD). Since ancient times, Chinese have cooked the tonic food which was well known as “bird's nest soup” [6]. The reported bioactivities and nutritional value of EBN included the potential for improving the abilities of mitogenic response and elevating the antivirus or lectin-binding activity and so on [7]. To date, this tonic food is still popular and savored by the Chinese community all over the world. Edible-nest swiftlets are only found in Southeast Asia [8]. In this area, Malaysia has emerged as the third largest EBN-producing country after Thailand and Indonesia.

Carbohydrates in the edible bird's nest are composed of sialic acid, which may include *N*-acetylneuraminic acid, galactosamine, glucosamine, or galactose [9]. *N*-acetylneuraminic acid is a nonreducing sugar residue in glycol proteins and mucins. Wang et al. [10] determined the content of *N*-acetylneuraminic acid in EBN to be 7.1%–9.0% by chromatography on Alltima C18 column and concluded that it constituted 99% of the total sialic acid. Former studies have showed that EBN can improve the immunity or depress the influenza virus by the activities of sialic acid [11]. Besides the above functions, sialic acid can also play an important role in the development of mammals' brain and intelligence. It was reported that dietary sialic acid supplementation could improve learning and memory abilities and increase its content inside calvarium in piglets [10]. Moreover, the ganglioside induced by sialic acid can strengthen the long-term potentiation in hippocampi [12], leading to coordination with neurotrophin (NGF) in the brain [13]. However, little information exists about the effects of maternal administration of EBN on intelligence development in their offspring. It is therefore of great interest to define the transgenerational effects of EBN and the underlying mechanisms that may mediate these effects.

In the present study, we investigated the effects of EBN on cognitive function in the suckling offspring mice, together with sialic acid content in the milk. The contents of sialic acid in mouse milk were measured by HPLC methods. Learning and memory parameters of the offspring were evaluated by Morris water maze as a mean to determine the spatial memory. In addition, this study examined the effects of EBN on the activity of antioxidant indexes in the brains. Moreover, the activities of AChE and ChAT in palliums and hippocampi of cubs, together with BDNF and the numbers of dyed neurons, were assessed as well to verify the mediation of learning and memory processes by EBN administration in maternal mice.

## 2. Materials and Methods

### 2.1. Materials

The dried EBN was purchased from Kuantan, Malaysia, and provided by Xiamen Si Nong Co. Ltd. It was made into homogenate by the following method. Briefly, 9 g dried EBN was immersed in the distilled water for 5 h. Then the soaked EBN was stewed in the water for 20 min with the solid-liquid ratio at 1 : 20 (g/mL). After high-pressure sterilization, the EBN homogenate was prepared for the experiment.

Meanwhile, *N*-acetylneuraminic acid (purchased from Sigma-Aldrich Co. LLC), which constitutes the large majority compound in sialic acid, was dissolved in distilled water at the concentration of 0.5 mg/mL. Concentration of sialic acid was detected as described below. To verity the stability of EBN during storage, sialic acid was detected after 42 d storage. Additionally, BDNF antibody was purchased from Sigma Chemicals Co. Kits for measurement of SOD, MDA, AChE, and ChAT were purchased from Nanjing Jiancheng Bioengineering Institute (Nanjing, China).

### 2.2. Animals

ICR mice (7-8 weeks of age, ≥25 g, certificate no. SYXX2014-0005) were obtained from Fujian University of Traditional Chinese Medicine (Fujian, China). Housed in a regulated environment (20 ± 1°C) in the specific pathogen free (SPF) laboratory, every male mouse was accompanied by 3 female mice in each cage. The mice were grouped as listed in Table 1, with a 12 h light/dark cycle. Food and water were provided ad libitum. Before the start of the experiment, the mice lived in the laboratory for approximately 7 days to adapt to the experimental environment. The female mice with pessary would be regarded as pregnant mice. The original homogenate was prepared by 9 g dry EBN in every 182 g solution from the above method. There are 10 female mice in each group. The animals in the “high-dose” EBN group were orally administered with original homogenate. Dissolved by the distilled water, the homogenate was used to treat the female mice with the middle dose and the low dose at 4.5 g/182 g and 6.75 g/182 g (EBN/water), respectively. The mice administrated with *N*-acetylneuraminic acid were included in the positive control group. Every 10 free suckling mice from each group were sacrificed at the end of the lactation.

### 2.3. Assays for Free and Oligosaccharide-Bounded or Protein-Bounded Sialic Acid in Mouse Milk

#### 2.3.1. Detection of Free and Oligosaccharide-Bounded Sialic Acid

500 *μ*L of milk was accurately filled into the centrifuge tube, an equal volume of 10% trichloroacetic acid solution was added to make the protein precipitate, and the solution was sat inside the ice bath for 10 min after mixing. After centrifuging for 30 min, the supernatant was collected. Then the precipitate was washed with 500 *μ*L cold 5% trichloroacetic acid, followed by centrifuged (4°C, 3000 r/min) for 30 min. The supernatant was removed and mixed with the supernatant removed before. The mixed supernatant was added with an equal volume of 0.1 mol/L trifluoroacetic acid at 80°C and hydrolyzed for 30 min, and the mixture was cooled and filtered through a 0.22 *μ*m filter. The filtered solution was precisely removed for 90 *μ*L and added 10 *μ*L DMB at 50°C by light escaping for 150 min before detection.

#### 2.3.2. Detection of the Protein-Bounded Sialic Acid

The precipitate disposed above was added with 2 mL sulpho acid (0.5 mol/L) and hydrolyzed at 80°C for 120 min. After cooling, the mixture was filtered and washed with a 0.22 *μ*m filter. Then 90 *μ*L of the filtered solution was accurately removed, and 10 *μ*L 4,5-dimethyl-1,2-phenylenediamine (DMB) was added by light escaping for 150 min. The content was assayed based on the chromatographic condition.

Sialic acid content was analyzed on an HPLC (Agilent 1200) using a reversed phase Agilent HC-C18 column (4.5 × 250 mm, 5 *μ*m) with fluorescent detection (excitation wavelength at 373 nm and emission wavelength at 448 nm). A solvent system consisting of methanol, acetonitrile, and water solution (7 : 8 : 85) was used as mobile phase at a flow rate of 0.9 mL/min.

### 2.4. Morris Water Maze

Every 10 offspring mice (21 days old) from each group were given four days trials consecutively, to find a hidden 9 cm diameter black platform in the III quadrant located 2 cm below the water surface of a pool with 1.3 m diameter. For each trial, the mice entered the pool from different locations of the pool. If the mice did not find the platform within 60 s, they were placed on the platform and returned to their home cage after 10 s. The intertrial interval for each mouse was 5 min. On day 5, the platform was removed from the pool, and each mouse was tested by a spatial probe trial for 60 s. The times for crossing the former location of the platform and the length of time staying in the III quadrant of cubs were also recorded, analyzed, and compared among the groups. Data were recorded using the EthoVision automated tracking system (Noldus Information Technology).

### 2.5. Dissection and Collection of Tissues and Mouse Milk

#### 2.5.1. Collection of Mouse Milk

The milk of postpartum female mice was collected during the lactation period. Before the collection day, the female mice were separated from their corresponding offspring. In the next morning, the milk was collected and stored at −20°C for subsequent determination of sialic acid content.

#### 2.5.2. Collection of Cerebra Tissue

The suckling mice in all groups were weighed, and their cerebra tissues were isolated, weighed, and packed by tinfoil. Then the tissues were frozen quickly by liquid nitrogen and stored at −80°C.

#### 2.5.3. Isolation of Palliums and Hippocampi

After the cerebra were dissected, cut, and separated, the hippocampi were exposed and extracted after the isolation of palliums. Both palliums and hippocampi were stored at −80°C.

### 2.6. Assays of SOD and MDA Levels in the Cerebral Tissues

The levels of MDA in the cerebral tissues of the suckling mice were detected strictly in accordance with the instructions of the kits. The activities of SOD were performed by total superoxide dismutase (T-SOD) assay kit (hydroxylamine method) [14].

### 2.7. Assay of AChE and ChAT Levels

To determine the AChE and ChAT activities in both palliums and hippocampi in the offspring, the mice in all groups were decapitated after lactation period and the palliums and hippocampi were extracted. The activities of AChE and ChAT were estimated using assay kits provided by Nanjing Jiancheng Bioengineering Institute. The protocols of the kits were followed during the estimation procedure.

### 2.8. Brain-Derived Neurotrophic Factor (BDNF) Immunohistochemical Staining

#### 2.8.1. Preparation of Specimens

Isolated hippocampi were rinsed with normal saline and fixed with 4% polymethylene acid for 24 h. After rinsing in the water for several hours, the tissues were dehydrated by 70%, 80%, and 90% ethanol solution, respectively, and soaked in the solution consisting of ethanol and xylene in the same volume for 15 min, followed by soaking in xylene for 15 min. After that, the hippocampi were soaked in the solution consisting of xylene and paraffin wax in the equal volume for 15 min and then placed in paraffin wax for about 1 h. Then the tissues were paraffin-embedded and sectioned.

#### 2.8.2. Assay of BDNF in Hippocampi

The tissue slices were baked at 75°C for 1.5 h and placed in xylene for 10 min twice. After that, they were immersed in pure ethanol, 95% ethanol, 80% ethanol, and purified water for 5 min, respectively. Then they were put in the retrieval kit and added to the antigen retrieval solutions (citrate buffer solution). Furthermore, the slices were heated by autoclave until the appearance of the automatic deflation. After adding with the primary antibody solution (BDNF, 4°C, put for one night) and rinsing with PBS, rabbit/mouse secondary antibody solution was added to the slices and they were incubated at room temperature for 1 h. The slices were redyed with haematin for 3 min and dehydrated, coverslipped, and observed under microscopy observation at the different districts (DG/CA1/CA2/CA3) of the hippocampi.

### 2.9. The Neuron Count in Hippocampi

The tissue slices of hippocampi were treated with dewaxing and hydration. After washed twice with PBS, each slice was added with stain working solution until the whole tissue was covered. Then they were placed at room temperature for 40 min and washed with deionized water twice. The slices were dehydrated, transparent, coverslipped, and observed under the microscope.

### 2.10. Statistical Analysis

All values for each group were given as mean and standard deviation. The data were analyzed using one-way analysis of variance coupled with least significant difference in post hoc multiple comparisons using SPSS Statistics 20.0 (IBM, NY, USA). *P* value less than 0.05 was considered to be statistically significant.

## 3. Results

### 3.1. Detection and Stability of Sialic Acid in EBN during Storage

**Figure 1:**
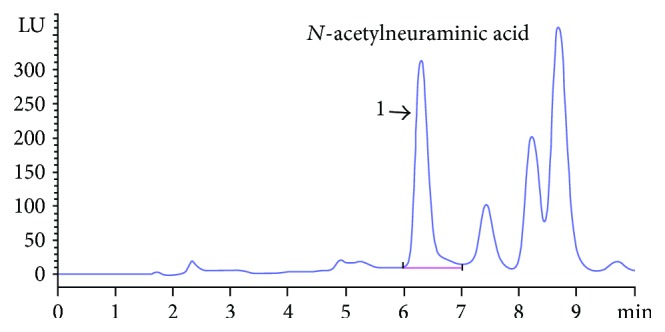
Analytical HPLC chromatogram of sialic acid in EBN. Peak 1 stands for *N*-acetylneuraminic acid compound of sialic acid.

As shown in Figure 1, the characteristic absorption peak of *N*-acetylneuraminic acid, which represented the large majority of sialic acid, was detected. HPLC chromatogram of NPA was complicated. It indicated major peak of *N*-acetylneuraminic acid showing identical retention time with the standard compound. Based on the comparison of peak areas with standard sample, the concentration of sialic acid in EBN homogenate was quantified to be 0.45 ± 0.05 g/100 mL. To verity the stability of EBN during storage, concentration of sialic acid was detected after 42 d storage. The detective value was 0.43 ± 0.08 g/100 mL, which showed no significant difference with the previous sample. It indicated that the symbolic compound in EBN did not degrade or change during its long-time storage.

### 3.2. Effects of EBN on Levels of the Sialic Acid in Mouse Milk

#### 3.2.1. Free and Oligosaccharide-Binding Sialic Acid (FSA)

**Figure 2:**
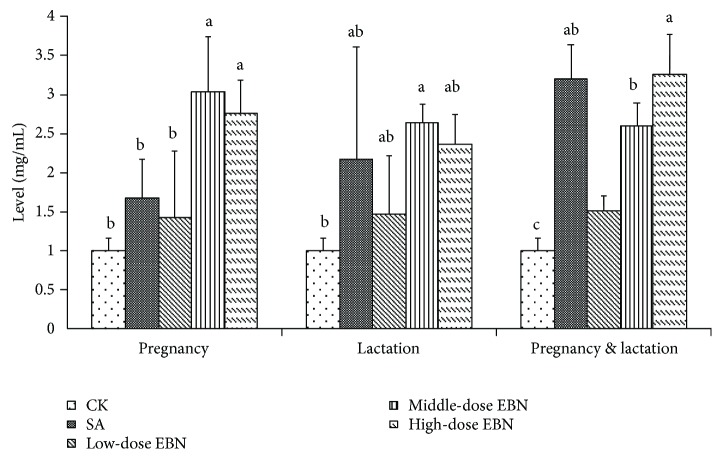
Comparison of free and oligosaccharide-binding sialic acid (FSA) level in mouse milk from each group of 10 female mice administrated with EBN. Significant differences were indicated by different letters for each group (*P* < 0.05).

Among the groups administrated with EBN or sialic acid during pregnancy, the levels of FSA in the high- and middle-dose groups were higher than those in the negative control or sialic acid groups. However, when the mice were administrated during lactation period, the levels in the middle-dose group were significantly increased, which showed no difference from the sialic acid group. Among the groups administrated with EBN or sialic acid during both lactation and pregnancy periods, the levels in the higher-dose group of EBN or the sialic acid group were stronger than those in the negative control group (Figure 2). These results indicated that maternal administration of EBN during pregnancy or lactation period can improve FSA in the milk, which will benefit the lactational offspring.

#### 3.2.2. Protein-Bounded Sialic Acid (PSA)

**Figure 3:**
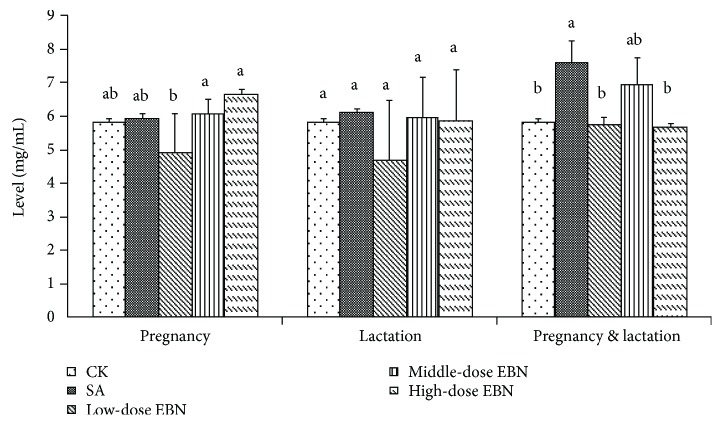
Comparison of protein-bounded sialic acid (PSA) level in mouse milk from each group of 10 female mice administrated with EBN. Significant differences were indicated by different letters for each group (*P* < 0.05).

When the groups were administrated with EBN or sialic acid during pregnancy, lactation, or the both periods, the levels of PSA had no significant difference comparing with the negative control group, indicating that EBN cannot improve the expression of PSA during these periods. However, PSA levels were significantly improved in the positive control group administrated with sialic acid during both pregnancy and lactation periods, as compared with the control group (Figure 3).

### 3.3. Effects of EBN on Learning Performance in Cubs

**Figure 4:**
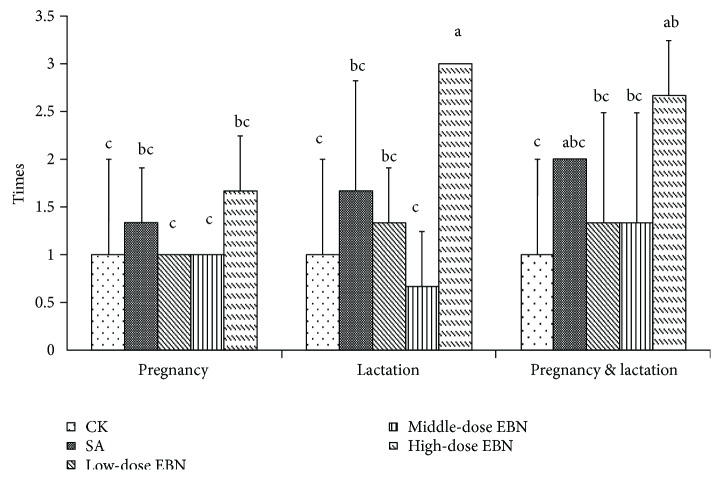
Times for which 10 cubs of each group crossed the target area after treated with edible bird's nest (EBN) homogenate in different doses. Significant differences were indicated by different letters for each group (*P* < 0.05).

As shown in Figure 4, feeding the maternal mice with high-dose EBN in lactation period could benefit learning performance of the cubs, which showed more times for crossing the target area. In contrast, the sulking cubs from the maternal mice administrated with EBN during pregnancy period showed no significant improvement on their crossing times compared with the control group.

**Figure 5:**
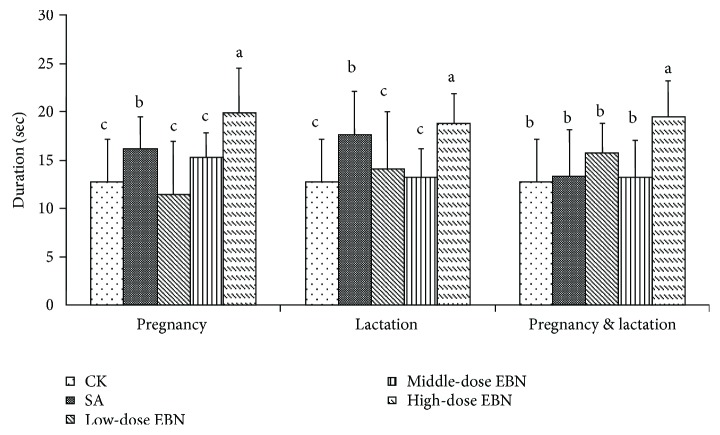
Time duration of 10 cubs from each group staying in the third quadrant after treated with edible bird's nest (EBN) homogenate. Significant differences were indicated by different letters for each group (*P* < 0.05).

The results of the duration time for the cubs staying in the third quadrant were demonstrated in Figure 5. The results showed that, along with the increase of EBN consumption for the maternal mice, the length of time for the cubs was also increased, depending on the dose (Figure 5). It suggested that high-dose EBN for the maternal mice during pregnancy or lactation period can benefit the memory function of the cubs, while the low- or middle-dose EBN for the maternal mice showed no significant improvements. And the groups treated during both periods followed a similar trend as the groups treated during lactation. Combined with the above results, it implied that high-dose EBN can significantly enhance memory function of the cubs.

### 3.4. Effects of EBN on the Cerebral Activities of SOD in Cubs

**Figure 6:**
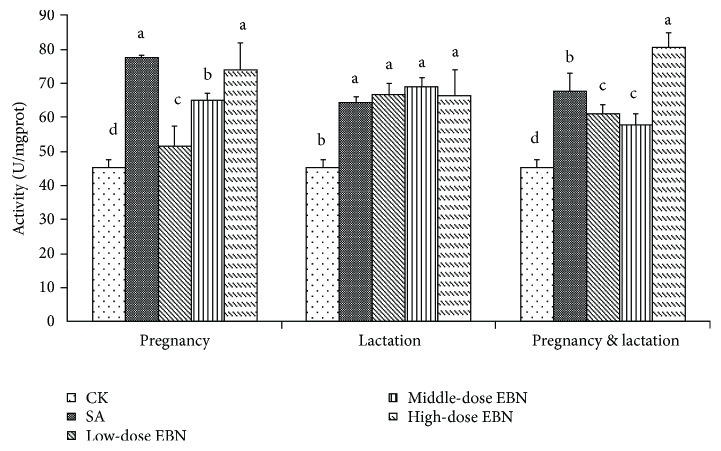
Comparison of superoxide dismutase (SOD) activity in brain tissue among the 10 weaning cubs from each group. Significant differences were indicated by different letters for each group (*P* < 0.05).

After weaning, the cerebral activities of SOD in the brain tissue of cubs from different groups were compared in Figure 6. Evidently, the activities of SOD in the negative control group were significantly lower than those in the other groups. Among the groups treated with EBN during pregnancy, the activities of SOD were dose-dependent and significantly higher than those in the control group. Among the groups treated during lactation, the activities were significantly higher than those in the negative control group and showed no marked difference mutually. However, when the groups were treated during both pregnancy and lactation periods, the group treated with high-dose EBN showed significantly higher activities than those in the other groups (Figure 6).

### 3.5. Effects of EBN on the Cerebral Levels of MDA in Cubs

**Figure 7:**
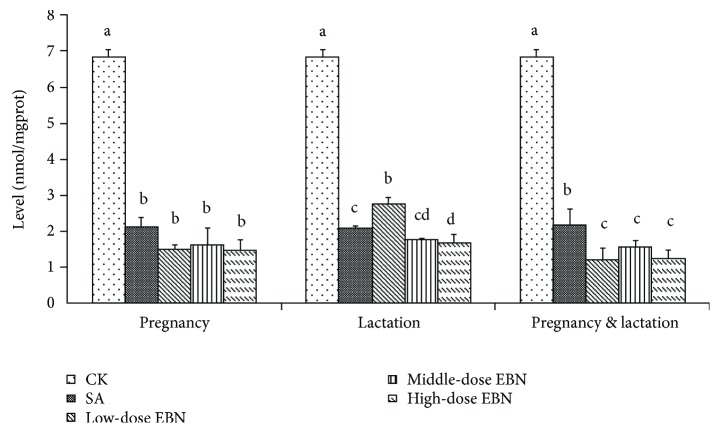
Comparison of malondialdehyde (MDA) activity in brain tissue among the 10 weaning cubs from each group. Significant differences were indicated by different letters for each group (*P* < 0.05).

The effects of EBN on MDA activities in brain tissue among the weaning cubs were indicated in Figure 7. It showed that MDA activities in brain tissue were significantly reduced in different treated periods after administration with EBN, which revealed that EBN may help to improve antioxidant condition in the offspring brain tissue. Particularly, the more desirable effect was found in groups with the intake of EBN happened during both pregnancy and lactation periods (Figure 7).

### 3.6. Effects of EBN on the Activities of the ChAT and AChE in Palliums and Hippocampi of Cubs

**Figure 8:**
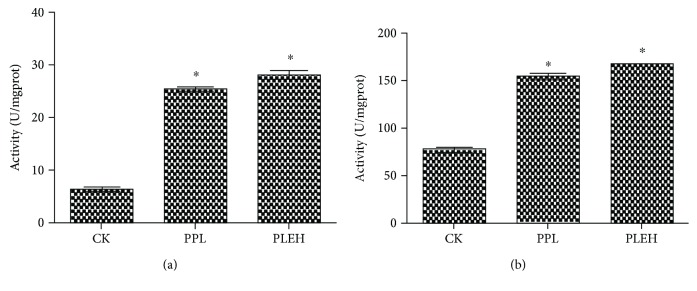
Comparison of choline acetyltransferase (ChAT) activities for hippocampi (a) or palliums (b) from suckling offspring among the negative control (CK) group, pregnancy and lactation sialic acid control (PPL) group, and pregnancy and lactation EBN high-dose (PLEH) group. Significant differences were indicated by the symbol “*∗*” for each group (*P* < 0.05).

In order to investigate the relation between the development of neurons in palliums or hippocampi of cubs and their intelligence, the activities of ChAT and AChE in palliums and hippocampi of cubs from the group administrated with high-dose EBN during both pregnancy and lactation were detected. The results showed that, compared with the cubs from the negative control group, there was significant increase in the activity of ChAT, while it showed no significant differences with that in the positive control group (Figure 8). The variation tendency of ChAT in palliums was quite similar with that in hippocampi, which indicated that EBN can positively impact on learning performance in cubs by increasing ChAT activities.

**Figure 9:**
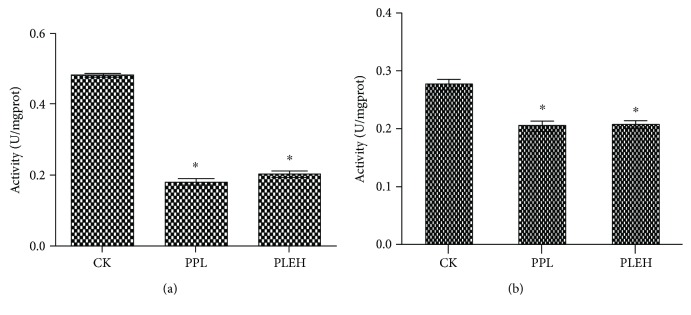
Comparison of acetylcholinesterase (AChE) activities for hippocampi (a) or palliums (b) from suckling offspring among the CK, PPL, and PLEH groups. Significant differences were indicated by the symbol “*∗*” for each group (*P* < 0.05).

AChE can hydrolyze the neurotransmitter acetylcholine at neuromuscular junctions, thus terminating signal transmission in the brain. The major form of AChE found in the brain, muscle, and other tissues is the hydrophilic species, which forms disulfide-linked lipid-containing subunits to degrade memorial signaling molecule acetylcholine. As shown in our results, AChE activities from the brain tissue of the group PLEH were significantly reduced compared with the control group (Figure 9). Thus, maternal administration of EBN may help to improve the psychopathology as well as behavior in their offspring.

### 3.7. Immunohistochemical Staining of BDNF in the Hippocampus of Cubs

**Figure 10:**
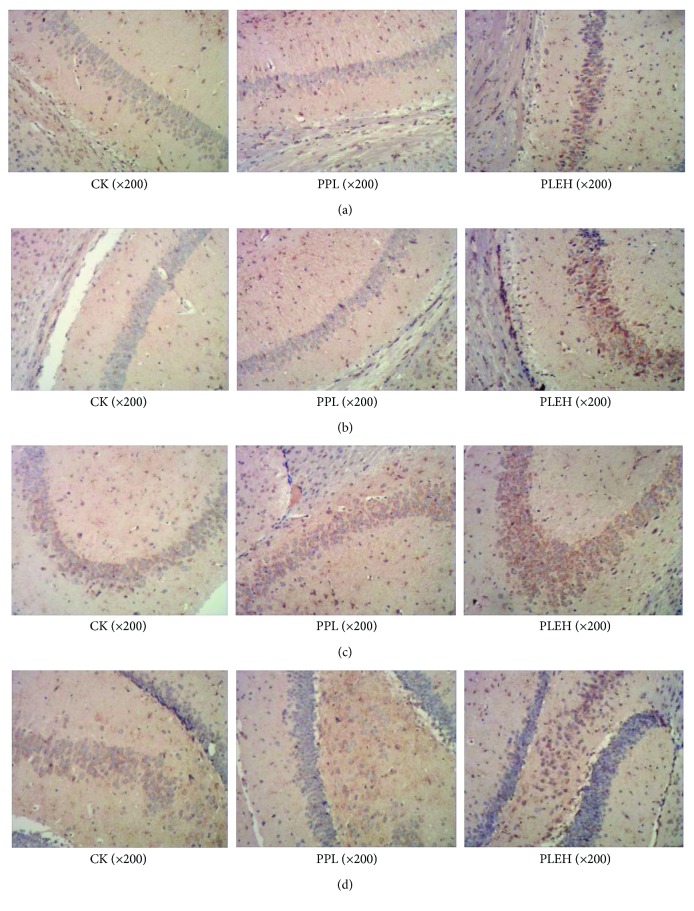
(a) Expressing situation of brain-derived neurotrophic factor (BDNF) in CA1 district of cubs' hippocampi. (b) Expressing situation of BDNF in CA2 district of cubs' hippocampi. (c) Expressing situation of BDNF in CA3 district of cubs' hippocampi. (d) Expressing situation of BDNF in DG district of cubs' hippocampi.

To investigate the effect of maternal administration of EBN on hippocampal neurotrophin levels in the offspring, we next examined the expression of BDNF in isolated hippocampi of different groups of the cubs. According to the immunohistochemical staining test, in CA1 and CA2 regions, the levels of BDNF from the high-dose group were significantly higher than those from the negative control and positive control groups. However, no significant difference was found between the sialic acid-treated group and the control group. In CA3 and DG regions, the levels in the positive control group were significantly higher than those in the negative control group, but there were no significant differences between the positive control group and the high-dose group (Figure 10). Thus, the levels of BDNF in CA1, CA2, and CA3 regions in hippocampi of cubs were able to be enhanced after the intake of EBN by the maternal mice, while administration of sialic acid in the maternal mice may help to increase the levels of BDNF in CA3 and DG regions.

These results demonstrated that EBN administrated in maternal mice can increase hippocampal BDNF production of the offspring and the BDNF variation trend paralleled the increase in their learning performance.

### 3.8. Neuron Count in Hippocampi of Cubs

**Figure 11:**
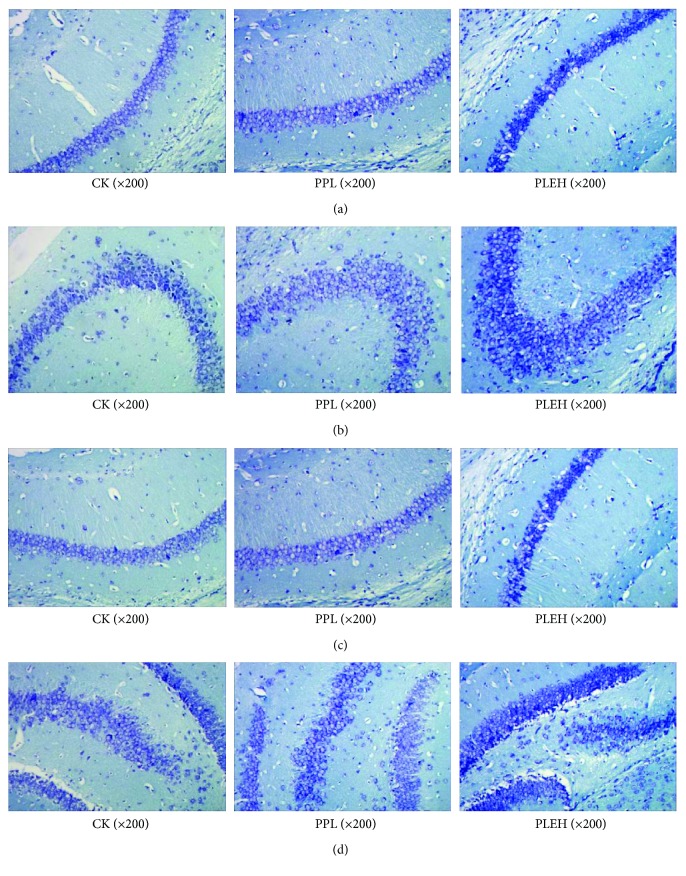
(a) Situation of Nissl body staining in CA1 district of cubs' hippocampi. (b) Situation of Nissl body staining in CA2 district of cubs' hippocampi. (c) Situation of Nissl body staining in CA3 district of cubs' hippocampi. (d) Situation of Nissl body staining in DG district of cubs' hippocampi.

In CA1, CA3, and DG regions, the numbers of dyed neurons in the EBN-administrated or positive control groups were significantly higher than those in the negative control group. However, in CA2 region, the differences between the PPL and CK groups were not remarkable (Figure 11). These data indicate that EBN-administrated offspring show increased levels of hippocampal BDNF and these offspring also show stronger dendritic differentiation of new hippocampal neurons during early postnatal development.

## 4. Discussion

As a product of physiological activity, breast milk has various nutrients which are essential for the growth and development of newborn individuals. It has been documented that maternal diet and metabolic function can affect brain development and function in their offspring [15]. Sialic acid, which is a derivative of a class of neuraminic acids widely found in the brain and milk of mammals, is an important ingredient to constitute the glycoprotein and glycolipid. One of the main sources for sialic acid is breast milk, existing in the form of free, oligosaccharide-binding, and protein-bounded. As a natural brain nutrient, sialic acid can promote infant brain memory and mental development. Animal experiments have proved that, with the increasing intake of sialic acid, the ability to complete the maze learning task was also enhanced, indicating that the intake of exogenetic sialic acid may play an important role on the brain development of cubs [16]. Unsleber et al. [17] found that abnormal behaviors in malnourished rats were decreased by giving intraperitoneal injection of *N*-acetylneuraminic acid repeatedly, concluding that there is a certain relationship between its behavior and *N*-acetylneuraminic acid intake.

EBN is derived from the saliva of certain types of swiftlet. It is consumed in many regions all over the world for its nutritional and medicinal values. It has been demonstrated to beneficially affect neural health and intelligence development. Sialic acid, one of the main nutrients from EBN, has been reported to constitute 9% total dry matter [18]. In the former research, Yu-Qin et al. [19] conducted the detection of several neuraminic acids (*N*-acetyl-d-galactosamine, *N*-acetyl-d-glucosamine, and *N*-acetylneuraminate) and confirmed that they are the main components for sialic acid from genuine EBN.

Our research showed that administration with EBN during pregnancy or both pregnancy and lactation periods can enhance the levels of free and oligosaccharide-binding sialic acid in mouse milk. It suggested that the maternal mice were able to obtain more sialic acid during the prenatal period, which may positively affect the brain development of their offspring. In terms of spatial learning performance in Morris water maze test, matching the results from assays of sialic acid levels, the cubs whose female parental generation individuals were orally administered high-dose EBN homogenate during pregnancy or both pregnancy and lactation periods exhibited better memory function and level of intelligence. These results indicate that maternal administration of EBN can improve the offspring's cognitive performance. Studies in humans have also shown that the maternal who own good nutrition during their pregnancy scores higher on the orientation and state regulation [20]. In our study, the Morris water maze test was employed to assess the persistent effect of maternal administration of EBN on the offspring's cognitive performance. Morris water maze test is a widely used experimental method for studying the spatial learning ability of rodents. Due to the stability and reliability of this method, it can be used in many experimental models to evaluate the cognitive performances [21] and may avoid the effect of difference among individuals. Many scholars have also supported that there must be a critical relation between the spatial learning ability of animals in the water maze experiment and the structure of the hippocampus in the brain [22, 23].

Our study also showed that EBN cannot only alleviate oxidative stress damage, but also improve the level of intelligence. As the most important oxidative stress indexes, the cerebral levels of MDA and SOD were determined in our research. It has been reported in the past study that EBN may attenuate high-fat-diet-induced oxidative stress [24]. Our results further approved that cerebral SOD activities in cubs can be strengthened by feeding their female parental generation during pregnancy with EBN homogenate (dose-dependent). The highest SOD activity was observed in the group fed with high-dose EBN homogenate during both pregnancy and lactation periods. On the other hand, all the groups treated with EBN or sialic acid have a significantly lower levels of MDA than the negative control group, while the groups fed with EBN during both pregnancy and lactation periods also had a significantly lower levels of MDA than their sialic acid counterpart. Thus, we can partly conclude that the improved intelligence level of EBN may be correlative with the reduced antioxidant status in brain tissue of the cubs.

Clinical and biochemical studies have found that insufficient ChAT activity and overexpression of AChE activity can easily lead to the decrease of cerebral acetylcholine content, leading to the dysfunction of central nervous system, which has a negative impact on learning, memory, and cognitive functions [25]. In this study, the activities of ChAT in the high-dose group were significantly improved compared with those in the negative control group. And the activities of AChE in the high-dose group were significantly reduced as compared with those in the negative control group. Therefore, high levels of sialic acid in breast milk can increase the brain's acetylcholine content by increasing ChAT activity and decreasing the activity of AChE in the palliums or hippocampi of the cubs, which were highly related to the improvement of learning and memory functions in the offspring.

Hippocampus is an important brain area for learning and memory, where neurogenesis (formation of new neurons) occurs throughout life and decreases substantially with ageing [26]. As an important place for higher neurological activity, hippocampus is closely linked with the abilities of learning or memory, while learning ability and the number of neurons in the hippocampus of the brain are also highly connected [27]. Neurogenesis occurs continuously in the hippocampal dentate gyrus throughout life. It is the process by which new neurons are generated. Neurogenesis comprises a complex process, beginning with the division of a progenitor cell and ending with the existence of functionally fully integrated new neurons. These new neurons undergo typical morphological differentiation and maturation and then integrate into the existing hippocampal circuits [28]. Our results showed that in different regions (CA1, CA2, CA3, and DG) of the hippocampus, the numbers of dyed neurons in the EBN-treated group were significantly increased compared with those in the control group, which suggested that the learning capacity was improved as the number of the neurons increased. Thus, increasing new neurons can further explicate the effect of EBN on improving learning performance of the offspring. Moreover, the maturation and integration of new neurons into the hippocampal circuits are highly complex and dynamic processes, in which BDNF has been suggested to play important roles during early neural development, including neuronal growth, plasticity, survival, and protection [29]. BDNF is widely expressed in hippocampus of individual brains and is extremely important for the survival of basal ganglia [30], other neurons, and synaptic plasticity [31]. Hippocampal BDNF level may be positively related to learning and memory efficiencies [32]. The present data indicate that PLEH offspring show increased levels of hippocampal BDNF and these offspring can also show outstanding dendritic differentiation of new hippocampal neurons during early postnatal development. As a neurotrophic factor, BDNF maintains the survival of neurons and promotes their differentiations. Our results further confirm the conclusion that BDNF can induce mitochondrial biogenesis in newly generated neurons which are fundamental to the long-lasting changes at synapses [33]. Furthermore, evidences have also proved that BDNF plays a critical role in learning and memory. It has been shown that the learning and memory dysfunctions in Morris water maze test occurred after the BDNF gene was knocked out [34]. Hence, we can confer that elevated learning ability in offspring from EBN administration of the maternal mice was related with their improvement of hippocampal BDNF.

In terms of BDNF content in hippocampi of cubs, BDNF in the regions of hippocampi (CA1/CA2/CA3) of the cubs from the high-dose EBN group was significantly higher than that from the negative control group. It implied that the intake of EBN by female mice during pregnancy and lactation periods can promote the synthesis of BDNF and facilitate the differentiation and growth of neurons. From these data, it can be postulated that BDNF increased by EBN administration during perinatal period may be one mechanism for the improvement of hippocampal neurogenesis during postnatal development.

In addition, it is known that BDNF is negatively correlated to oxidative stress [30]. Previous data suggested that oxidative stress and peroxidized lipid products were the main mechanisms for the reduction in the hippocampal BDNF and these factors would transiently influence BDNF production during the early postnatal development of the offspring [35]. Interestingly, our research showed that maternal administration of EBN can help to improve expression of BDNF in hippocampi of cubs, which seems to be highly correlated with a decrease in oxidative load in the brain tissues. Taken together, we suggest that the reduction of hippocampal oxidative marker in adulthood of the rats from maternal mice fed with EBN may probably occur via high hippocampal BDNF.

## 5. Conclusion

We proved the idea that maternal administration of EBN during the pregnancy and lactation periods can enhance the levels of sialic acid in maternal mouse milk, which was associated with the improvement of spatial learning performances, the increasing of ChAT or SOD activities, and the decreasing of AChE or MDA activities in palliums and hippocampi of their offspring. In addition, EBN administration in maternal mice can increase the levels of BDNF and boost the growth and differentiation of neurons among their generations. This is the first study examining the effects of maternal EBN administration on the learning and memory of the offspring. Although our research has proved that maternal administration of EBN may enhance the brain functions of the offspring, further studies should be directed towards investigating the changes of the cerebral nerve signal pathway. Our results may be useful for further studies aiming for nutrition treatment in the maternal administration for the intelligence development of the offspring.

## Figures and Tables

**Table 1:** Details of grouping and oral administration of the test substances.

Group number	Group name	Test substance	Concentration	Period of the treatment	Length of treated time (d)
1	Negative control (CK)	Normal saline	/	Pregnancy and lactation	42
2	Positive pregnancy control (PP)	*N*-acetylneuraminic acid	0.5 mg/mL	Pregnancy	21
3	Positive lactation control (PL)	*N*-acetylneuraminic acid	0.5 mg/mL	Lactation	21
4	Positive pregnancy and lactation control (PPL)	*N*-acetylneuraminic acid	0.5 mg/mL	Pregnancy and lactation	42
5	Pregnancy and EBN low dose (PEL)	EBN homogenate	4.5 g/182 g	Pregnancy	21
6	Pregnancy and EBN middle dose (PEM)	EBN homogenate	6.75 g/182 g	Pregnancy	21
7	Pregnancy and EBN high dose (PEH)	EBN homogenate	9 g/182 g	Pregnancy	21
8	Lactation and EBN low dose (LEL)	EBN homogenate	4.5 g/182 g	Lactation	21
9	Lactation and EBN middle dose (LEM)	EBN homogenate	6.75 g/182 g	Lactation	21
10	Lactation and EBN high dose (LEH)	EBN homogenate	9 g/182 g	Lactation	21
11	Pregnancy and lactation EBN low dose (PLEL)	EBN homogenate	4.5 g/182 g	Pregnancy and lactation	42
12	Pregnancy and lactation EBN middle dose (PLEM)	EBN homogenate	6.75 g/182 g	Pregnancy and lactation	42
13	Pregnancy and lactation EBN high dose (PLEH)	EBN homogenate	9 g/182 g	Pregnancy and lactation	42

All the female mice from each group were treated with 0.5 mL/d *N*-acetylneuraminic acid or EBN homogenate in their corresponding period. As for the negative control group, mice were treated with saline (0.5 mL/d).
